# Supplementary material for: L-threonine promotes healthspan by expediting ferritin-dependent ferroptosis inhibition in C. elegans
Source: Nat Commun. 2022 Nov 2;13:6554. doi: 10.1038/s41467-022-34265-x (PMC9628521; doi:10.1038/s41467-022-34265-x)
Supplement: Supplementary file 1 — Supplementary Information [file 41467_2022_34265_MOESM1_ESM.pdf]

Supplementary Information

L-threonine promotes healthspan by expediting ferritin-dependent ferroptosis inhibition in *C. elegans*

Juewon Kim[1,*], Yunju Jo[2], Donghyun Cho[1,] Dongryeol Ryu[2,*]

[1]Basic Research & Innovation Division, Amorepacific R&D Center, Yongin, Korea

[2]Department of Molecular Cell Biology, Sungkyunkwan University School of Medicine, Suwon, Korea.

*Correspondence: Juewon Kim (kimjw@amorepacific.com) and Dongryeol Ryu (freefall@skku.edu)

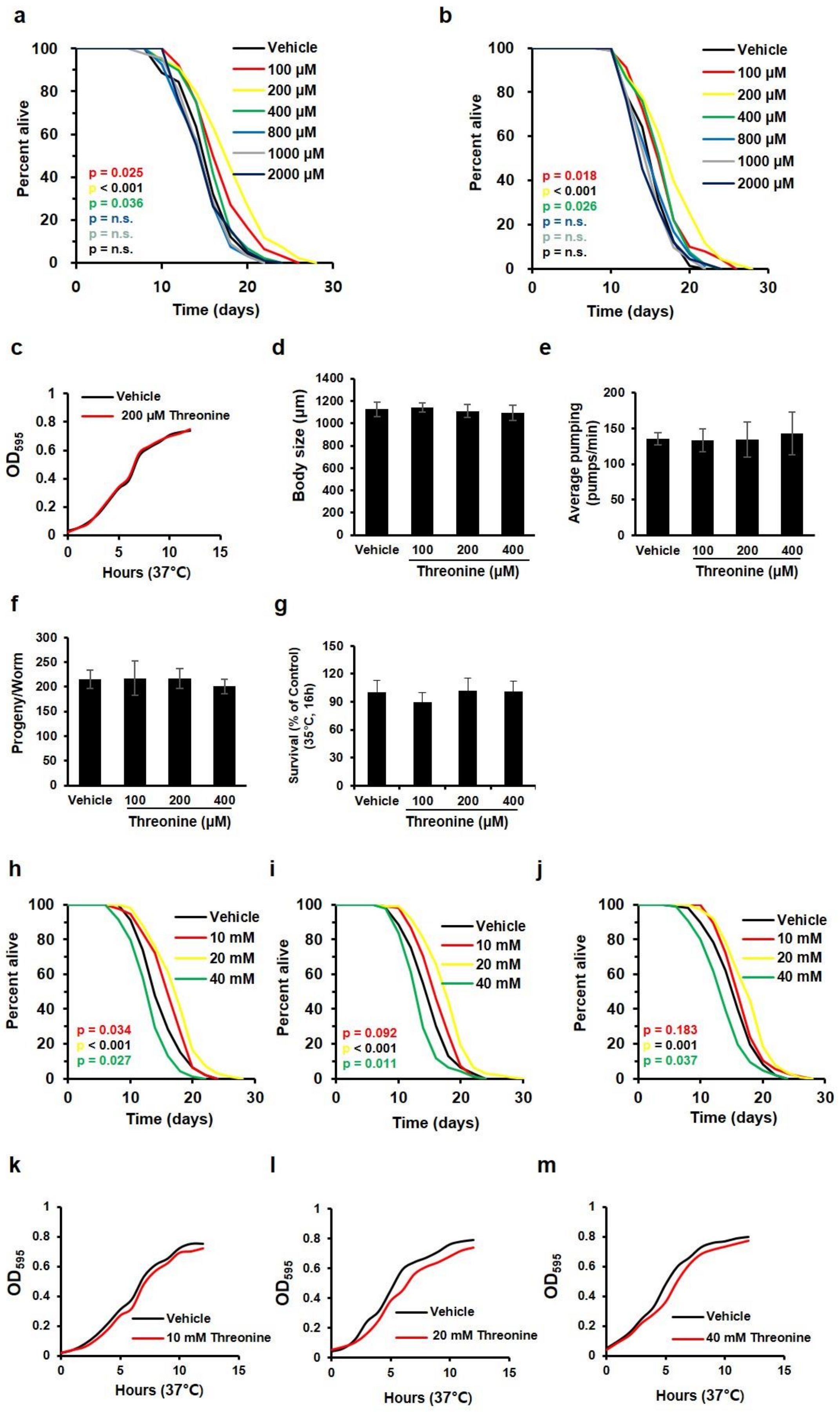
a
Percent alive
Time (days)
Vehicle
100 µM
200 µM
400 µM
800 µM
1000 µM
2000 µM
p = 0.025
p < 0.001
p = 0.036
p = n.s.
p = n.s.
p = n.s.
b
Percent alive
Time (days)
Vehicle
100 µM
200 µM
400 µM
800 µM
1000 µM
2000 µM
p = 0.018
p < 0.001
p = 0.026
p = n.s.
p = n.s.
p = n.s.
c
OD595
Hours (37°C)
Vehicle
200 µM Threonine
d
Body size (µm)
Vehicle
100
200
400
Threonine (µM)
e
Average pumping (pumps/min)
Vehicle
100
200
400
Threonine (µM)
f
Progeny/Worm
Vehicle
100
200
400
Threonine (µM)
g
Survival (% of Control) (35°C, 16h)
Vehicle
100
200
400
Threonine (µM)
h
Percent alive
Time (days)
Vehicle
10 mM
20 mM
40 mM
p = 0.034
p < 0.001
p = 0.027
i
Percent alive
Time (days)
Vehicle
10 mM
20 mM
40 mM
p = 0.092
p < 0.001
p = 0.011
j
Percent alive
Time (days)
Vehicle
10 mM
20 mM
40 mM
p = 0.183
p = 0.001
p = 0.037
k
OD595
Hours (37°C)
Vehicle
10 mM Threonine
l
OD595
Hours (37°C)
Vehicle
20 mM Threonine
m
OD595
Hours (37°C)
Vehicle
40 mM Threonine

Supplementary Fig.1 | Threonine prolongs lifespan without diet or fertility changes. a-b, Survival curves depicted in Fig. 1a with additional replicates (p-values listed, log-rank test). **c**, Threonine does not alter the growth rate of the OP50 *E. coli*, which is the standard food source for nematodes. **d-g**, Effects of threonine versus the vehicle in animals regarding (**d)** body size ($p > 0.5$, $n = 6$), (**e**) average pumping ($p > 0.05$, Student's *t*-test, $n = 10$ worms $\times$ 3 assays each), (**f**) progeny ($p > 0.05$, Student's *t*-test, three independent measurement), and (**g**) thermotolerance ($P > 0.05$, Student's *t*-test, $n = 20$ worms $\times$ 9 measurements each). **h-j**, Survival curves of high concentration threonine (10, 20, and 40 mM) with additional repeats (p-values listed, log-rank test). Data of lifespan analysis are displayed in Supplementary Table 1. **k-m**, High concentration of threonine inhibited the growth rate of the nematode food source, OP50 strain. Error bars represent the mean ± s.d.

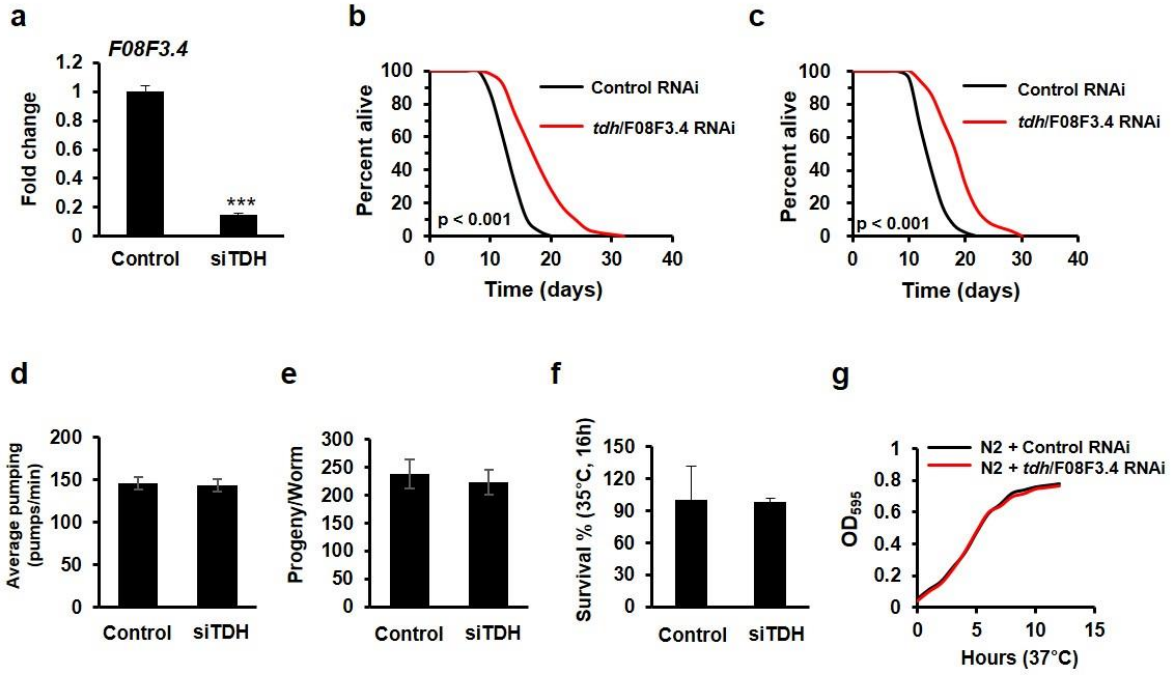

Supplementary Fig.2 | Downregulation of threonine dehydrogenase extends lifespan without diet or fertility changes. a, The efficiency of *tdh/F08F3.4* knockdown by RNAi was confirmed by quantitative RT-PCR (qRT-PCR) of the mRNA (71% decrease, ***p < 0.001, Student's *t*-test, three independent measurements). **b-c**, Survival curves depicted in Fig. 2k with additional replicates (p < 0.001, log-rank test). Survival data represented in Supplementary Table 1. **d-g**, Effects of *tdh/F08F3.4* RNAi versus control RNAi-treated animals regarding (**d**) average pumping (p = 0.748, Student's *t*-test, n = 10 worms × 3 assays each), (**e**) progeny (p = 0.487, Student's *t*-test, three independent measurement), and (**f**) thermotolerance (p = 0.92, Student's *t*-test, n = 20 worms × 9 measurements each). **g**, *tdh/F08F3.4* RNAi does not change the growth rate of OP50 *E. coli*. Error bars represent the mean ± s.d.

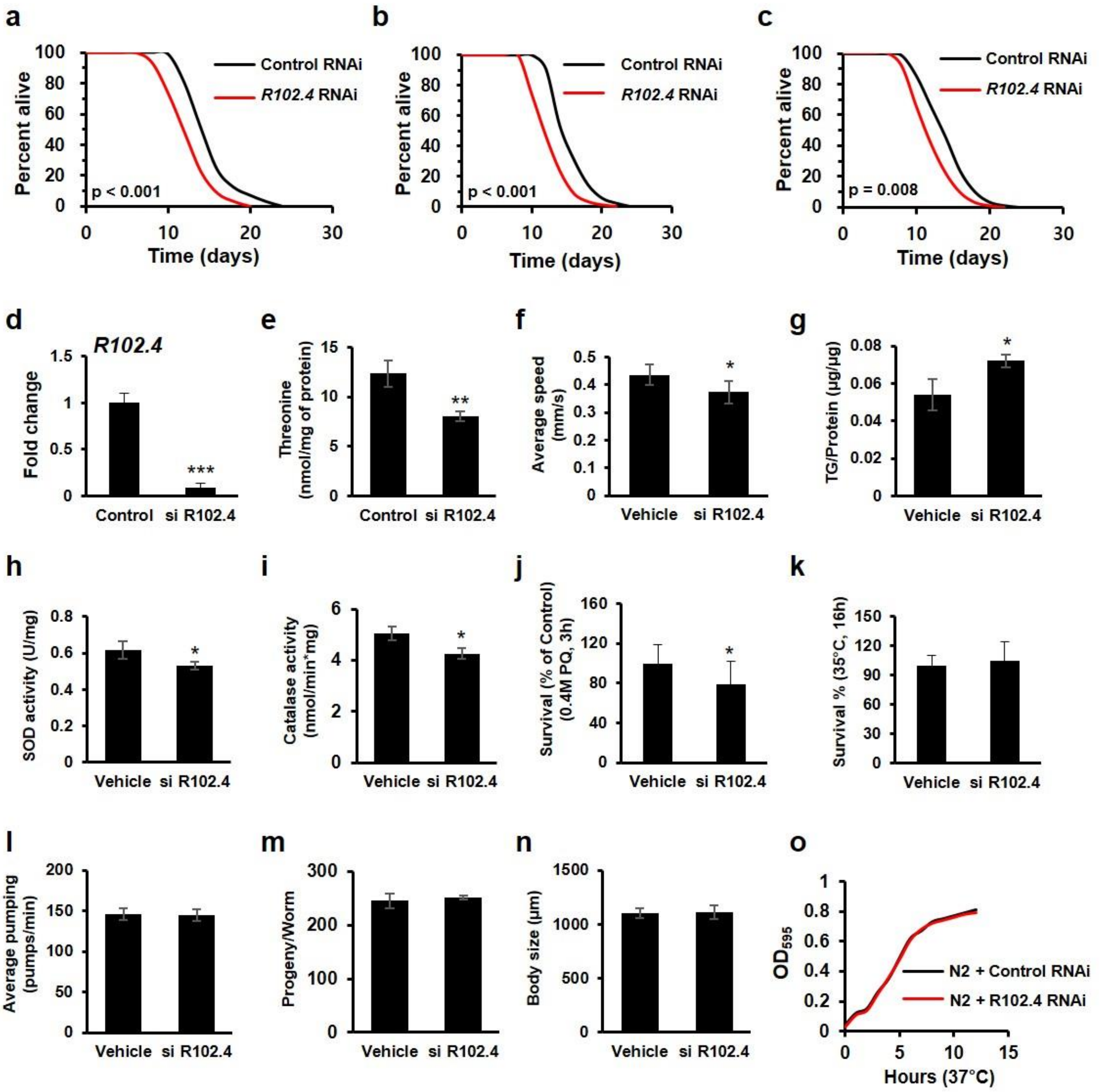
a
Percent alive
Control RNAi
R102.4 RNAi
p < 0.001
Time (days)
b
Percent alive
Control RNAi
R102.4 RNAi
p < 0.001
Time (days)
c
Percent alive
Control RNAi
R102.4 RNAi
p = 0.008
Time (days)
d
R102.4
Fold change
***
Control
si R102.4
e
Threonine (nmol/mg of protein)
**
Control
si R102.4
f
Average speed (mm/s)
*
Vehicle
si R102.4
g
TG/Protein (μg/μg)
*
Vehicle
si R102.4
h
SOD activity (U/mg)
*
Vehicle
si R102.4
i
Catalase activity (nmol/min*mg)
*
Vehicle
si R102.4
j
Survival (% of Control) (0.4M PQ, 3h)
*
Vehicle
si R102.4
k
Survival % (35°C, 16h)
Vehicle
si R102.4
l
Average pumping (pumps/min)
Vehicle
si R102.4
m
Progeny/Worm
Vehicle
si R102.4
n
Body size (μm)
Vehicle
si R102.4
o
OD595
N2 + Control RNAi
N2 + R102.4 RNAi
Hours (37°C)

Supplementary Fig.3 | Downregulation of threonine anabolic enzyme *R102.4* shortens lifespan and reduces healthspan. a-c, Survival curves of R102.4 RNAi versus control RNAi (black) with additional replicates ($p < 0.001$ or $p = 0.008$, log-rank test). Lifespan assay data are depicted in Supplementary Table 1. **d**, The efficiency of *R102.4* knockdown by RNAi was confirmed by quantitative RT-PCR (qRT-PCR) of the mRNA (89.9% decrease, ***$p < 0.001$, Student's t-test, 3 independent measurement). **e-o**, Effects of *R102.4* RNAi versus control RNAi regarding (**e**) threonine content (**$p = 0.006$, Student's *t*-test, n =3 worm pellets), (**f**) average speed (*$p = 0.021$, Student's *t*-test, n = 10-15 worms × 3 assays each), (**g**) triglyceride (TG) content (*$p = 0.025$, Student's *t*-test, n =3 worm pellets), (**h**) superoxide dismutase (SOD) activity (*$p = 0.05$, Student's *t*-test, n = 3 worm pellets), (**i**) catalase activity (**$p = 0.014$, Student's *t*-test, n = 3 worm pellets), (**j**) oxidative stress resistance (*$p = 0.049$, Student's *t*-test, n = 20 worms × 9 measurements each), (**k**) thermotolerance ($p = 0.726$, Student's *t*-test, n = 20 worms × 9 measurements each), (**l**) average pumping ($p = 0.852$, Student's *t*-test, n = 10 worms × 3 assays each), (**m**) progeny ($p = 0.498$, Student's *t*-test, three independent measurements), and (**n**) body size ($p = 0.906$, n = 6). (**o**) *R102.4* RNAi does not shift the growth rate of the OP50 *E. coli*. Error bars represent the mean ± s.d.

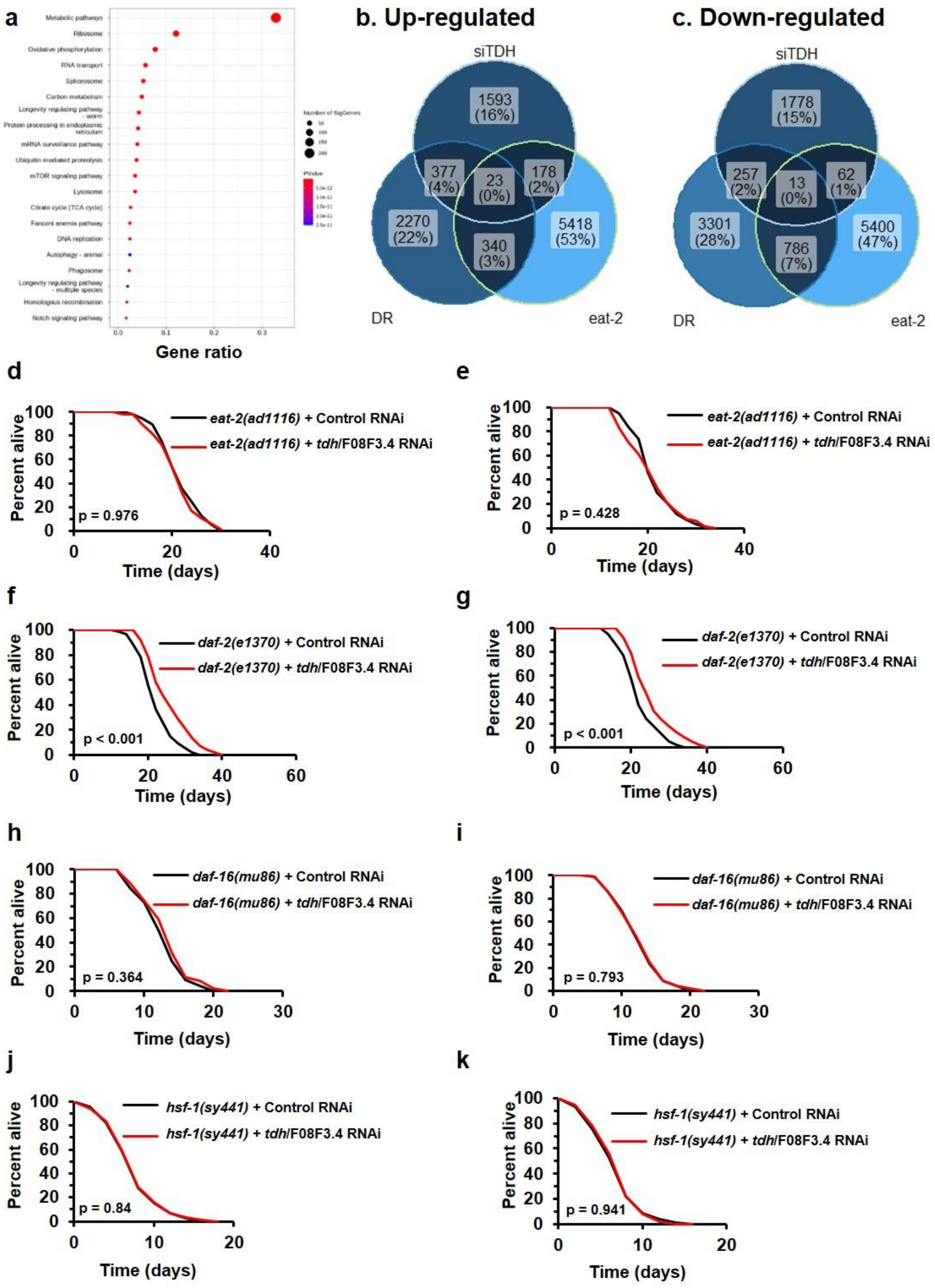
a
Metabolic pathways
Ribosome
Oxidative phosphorylation
RNA transport
Spliceosome
Carbon metabolism
Longevity regulating pathway - worm
Protein processing in endoplasmic reticulum
mRNA surveillance pathway
Ubiquitin mediated proteolysis
mTOR signaling pathway
Lysosome
Citrate cycle (TCA cycle)
Fanconi anemia pathway
DNA replication
Autophagy - animal
Phagosome
Longevity regulating pathway - multiple species
Homologous recombination
Notch signaling pathway
Gene ratio
b. Up-regulated
siTDH
1593 (16%)
377 (4%)
23 (0%)
178 (2%)
2270 (22%)
340 (3%)
5418 (53%)
DR
eat-2
c. Down-regulated
siTDH
1778 (15%)
257 (2%)
13 (0%)
62 (1%)
3301 (28%)
786 (7%)
5400 (47%)
DR
eat-2
d
eat-2(ad1116) + Control RNAi
eat-2(ad1116) + tdh/F08F3.4 RNAi
p = 0.976
Percent alive
Time (days)
e
eat-2(ad1116) + Control RNAi
eat-2(ad1116) + tdh/F08F3.4 RNAi
p = 0.428
Percent alive
Time (days)
f
daf-2(e1370) + Control RNAi
daf-2(e1370) + tdh/F08F3.4 RNAi
p < 0.001
Percent alive
Time (days)
g
daf-2(e1370) + Control RNAi
daf-2(e1370) + tdh/F08F3.4 RNAi
p < 0.001
Percent alive
Time (days)
h
daf-16(mu86) + Control RNAi
daf-16(mu86) + tdh/F08F3.4 RNAi
p = 0.364
Percent alive
Time (days)
i
daf-16(mu86) + Control RNAi
daf-16(mu86) + tdh/F08F3.4 RNAi
p = 0.793
Percent alive
Time (days)
j
hsf-1(sy441) + Control RNAi
hsf-1(sy441) + tdh/F08F3.4 RNAi
p = 0.84
Percent alive
Time (days)
k
hsf-1(sy441) + Control RNAi
hsf-1(sy441) + tdh/F08F3.4 RNAi
p = 0.941
Percent alive
Time (days)

Supplementary Fig.4 | Downregulation of threonine dehydrogenase alter metabolic process by DAF-16 and HSF-1-mediated mechanism. a, Functional annotation clustering for the DEGs from the volcano and smear plot of Fig. 3b-c ($p < 0.05$) determined by gene enrichment analysis using the KEGG database ($p < 0.05$). **b-c**, Venn analysis of transcripts that are regulated by *tdh/F08F3.4* RNAi (siTDH), dietary restriction (DR), and *eat-2* mutant (eat-2); (**b**) upregulated and (**c**) downregulated genes. **d-e**, Survival curves depicted in Fig. 3d with additional replicates (p-value listed, log-rank test). **f-g**, Survival curves depicted in Fig. 3e with additional replicates ($p < 0.001$, log-rank test). **h-i**, Survival curves depicted in Fig. 3f with additional replicates (p-value listed, log-rank test). **j-k**, Survival curves depicted in Fig. 3g with additional replicates (p-value listed, log-rank test). Survival data are presented in Supplementary Table 1.

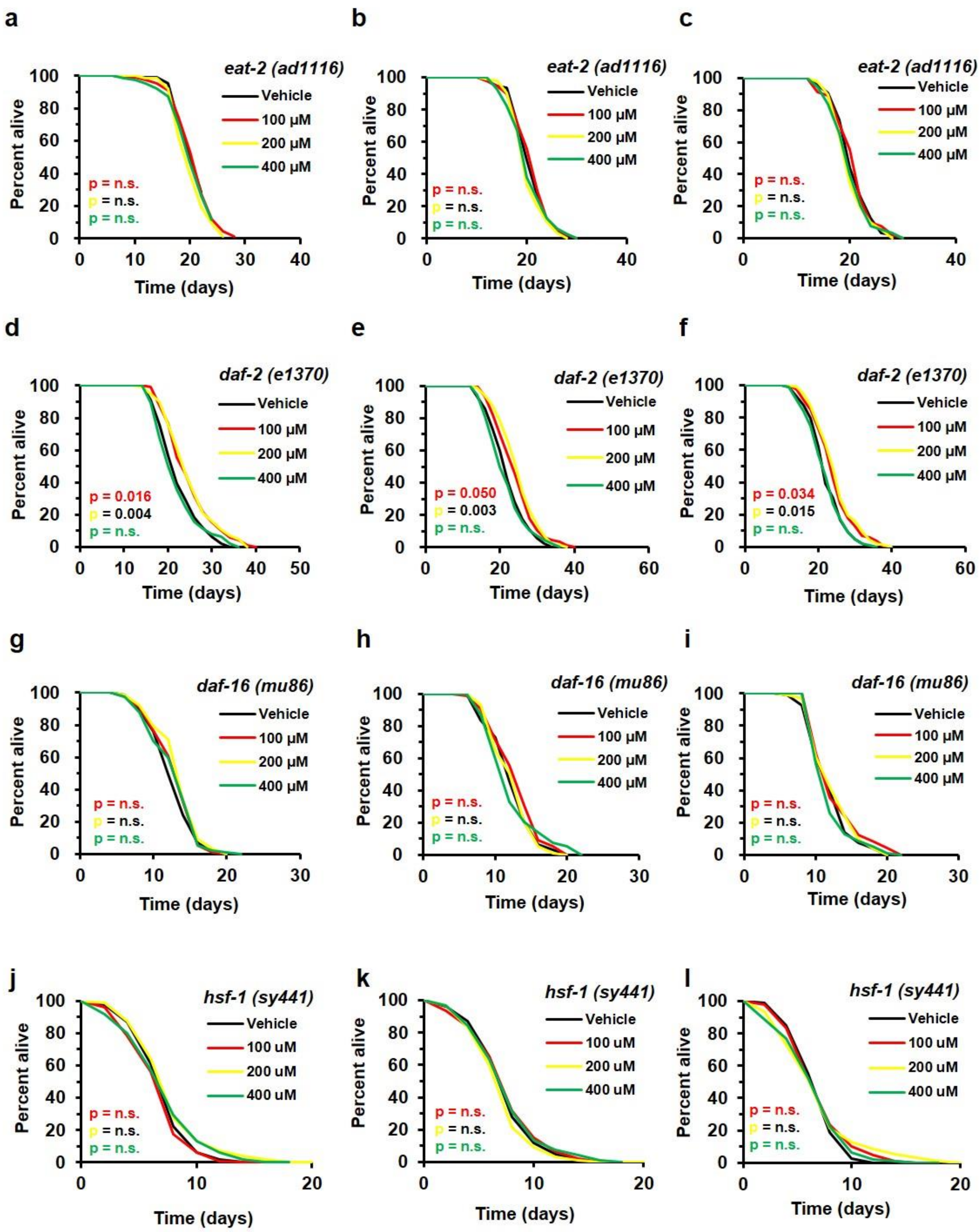
a
eat-2 (ad1116)
Vehicle
100 µM
200 µM
400 µM
p = n.s.
p = n.s.
p = n.s.
Percent alive
Time (days)
b
eat-2 (ad1116)
p = n.s.
p = n.s.
p = n.s.
c
eat-2 (ad1116)
p = n.s.
p = n.s.
p = n.s.
d
daf-2 (e1370)
p = 0.016
p = 0.004
p = n.s.
e
daf-2 (e1370)
p = 0.050
p = 0.003
p = n.s.
f
daf-2 (e1370)
p = 0.034
p = 0.015
p = n.s.
g
daf-16 (mu86)
p = n.s.
p = n.s.
p = n.s.
h
daf-16 (mu86)
p = n.s.
p = n.s.
p = n.s.
i
daf-16 (mu86)
p = n.s.
p = n.s.
p = n.s.
j
hsf-1 (sy441)
Vehicle
100 uM
200 uM
400 uM
p = n.s.
p = n.s.
p = n.s.
k
hsf-1 (sy441)
p = n.s.
p = n.s.
p = n.s.
l
hsf-1 (sy441)
p = n.s.
p = n.s.
p = n.s.

Supplementary Fig.5 | Threonine supplementation extends lifespan by a DAF-16 and HSF-1-mediated mechanism. a-l, Effects of L-threonine (100, 200, and 400 μM) versus the vehicle (black) on lifespan of **a-c**, *eat-2*(*ad465*), **d-f**, *daf-2*(*e1370*), **g-i**, *daf-16*(*m26*), and **j-l**, *hsf-1*(*sy441*) with additional replicates; colour coding is assigned to all subsequent panels. P-value and lifespan assay data summarized in Supplementary Table 1.

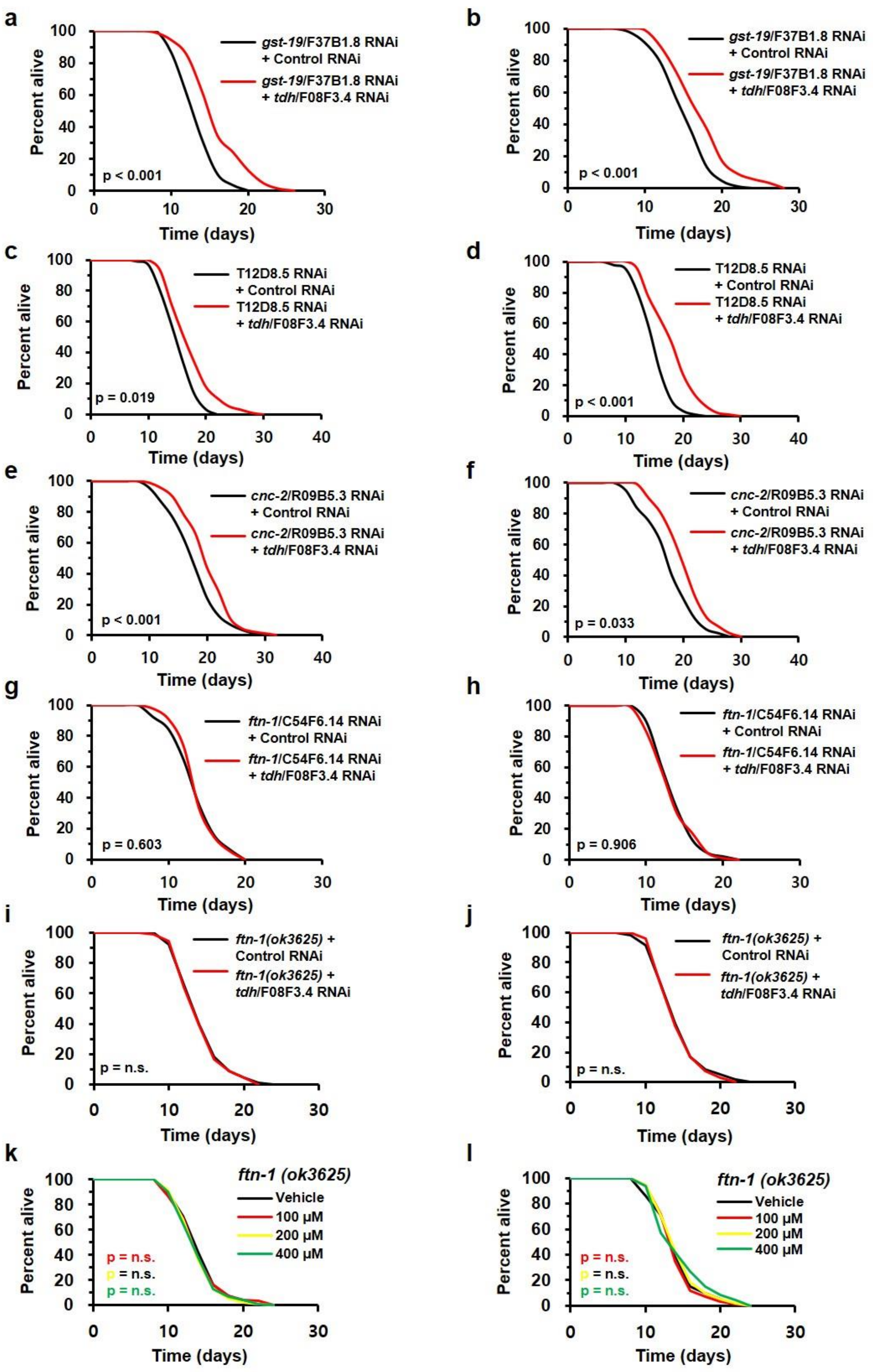

a
b
c
d
e
f
g
h
i
j
k
l
Percent alive
Time (days)
gst-19/F37B1.8 RNAi + Control RNAi
gst-19/F37B1.8 RNAi + tdh/F08F3.4 RNAi
p < 0.001
T12D8.5 RNAi + Control RNAi
T12D8.5 RNAi + tdh/F08F3.4 RNAi
p = 0.019
p < 0.001
cnc-2/R09B5.3 RNAi + Control RNAi
cnc-2/R09B5.3 RNAi + tdh/F08F3.4 RNAi
p < 0.001
p = 0.033
ftn-1/C54F6.14 RNAi + Control RNAi
ftn-1/C54F6.14 RNAi + tdh/F08F3.4 RNAi
p = 0.603
p = 0.906
ftn-1(ok3625) + Control RNAi
ftn-1(ok3625) + tdh/F08F3.4 RNAi
p = n.s.
ftn-1 (ok3625)
Vehicle
100 μM
200 μM
400 μM
p = n.s.
p = n.s.
p = n.s.

Supplementary Fig.6 | FTN-1 is necessary for threonine-mediated lifespan extension. a-b, Survival curves depicted in Fig. 4a with additional replicates. **c-d**, Survival curves depicted in Fig. 4b with additional replicates. **e-f**, Survival curves depicted in Fig. 4c with additional replicates. **g-h**, Survival curves depicted in Fig. 4d with additional replicates. **i-j**, Survival curves depicted in Fig. 4e with additional replicates. **k-l**, Survival curves depicted in Fig. 4f with additional replicates. P-values listed in figure panel and survival data are presented in Supplementary Table 1.

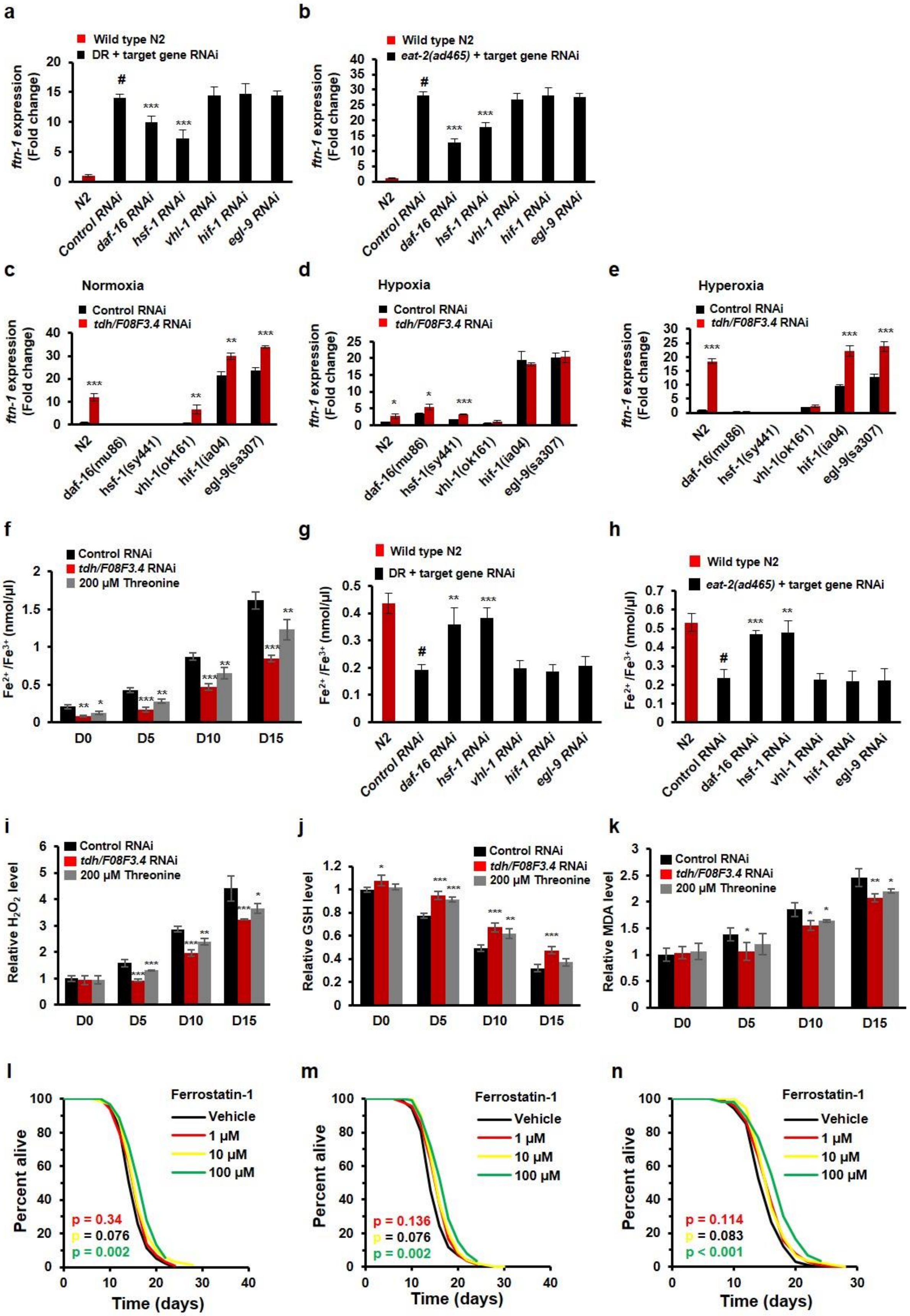
a
Wild type N2
DR + target gene RNAi
ftn-1 expression (Fold change)
N2
Control RNAi
daf-16 RNAi
hsf-1 RNAi
vhl-1 RNAi
hif-1 RNAi
egl-9 RNAi
b
Wild type N2
eat-2(ad465) + target gene RNAi
ftn-1 expression (Fold change)
c
Normoxia
d
Hypoxia
e
Hyperoxia
Control RNAi
tdh/F08F3.4 RNAi
ftn-1 expression (Fold change)
N2
daf-16(mu86)
hsf-1(sy441)
vhl-1(ok161)
hif-1(ia04)
egl-9(sa307)
f
Control RNAi
tdh/F08F3.4 RNAi
200 μM Threonine
Fe2+/Fe3+ (nmol/μl)
D0
D5
D10
D15
g
Wild type N2
DR + target gene RNAi
Fe2+/Fe3+ (nmol/μl)
h
Wild type N2
eat-2(ad465) + target gene RNAi
Fe2+/Fe3+ (nmol/μl)
i
Relative H2O2 level
j
Relative GSH level
k
Relative MDA level
l
m
n
Ferrostatin-1
Vehicle
1 μM
10 μM
100 μM
Percent alive
Time (days)
p = 0.34
p = 0.076
p = 0.002
p = 0.136
p = 0.076
p = 0.002
p = 0.114
p = 0.083
p < 0.001

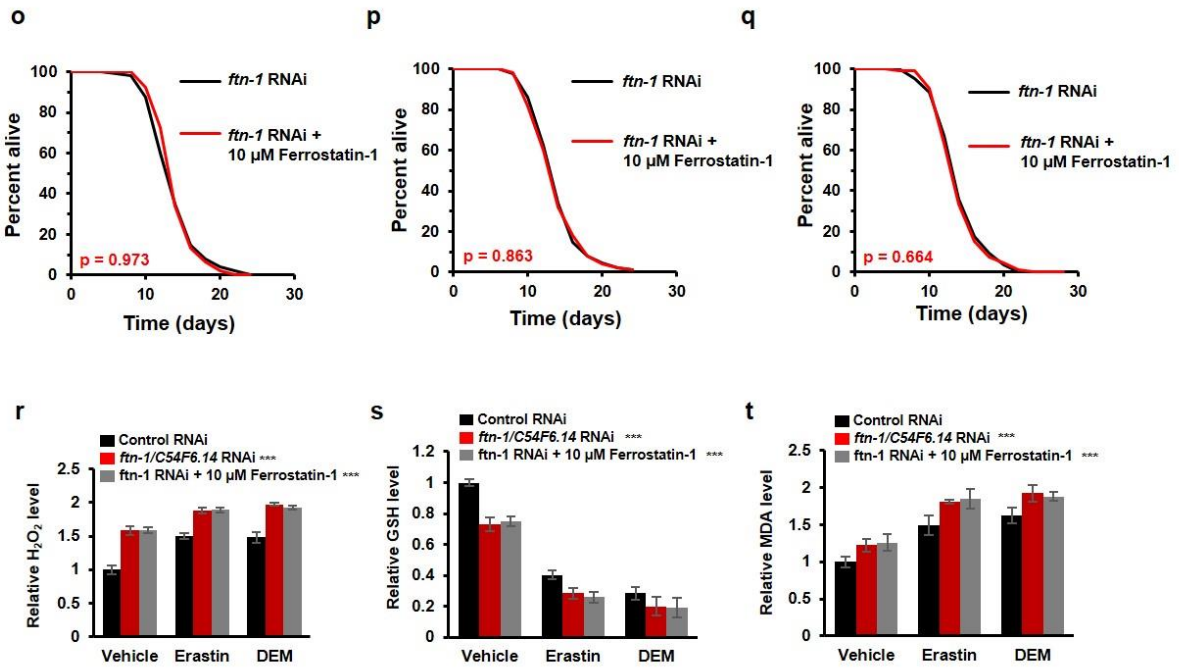
o
p
q
Percent alive
Time (days)
ftn-1 RNAi
ftn-1 RNAi + 10 μM Ferrostatin-1
p = 0.973
p = 0.863
p = 0.664
r
s
t
Control RNAi
ftn-1/C54F6.14 RNAi ***
ftn-1 RNAi + 10 μM Ferrostatin-1 ***
Relative H2O2 level
Relative GSH level
Relative MDA level
Vehicle
Erastin
DEM

Supplementary Fig.7 | Increased threonine attenuates ferroptosis by abrogating ROS, lipid peroxidation, and GSH depletion. a-b, Expression levels of *ftn-1* at **a**, DR, or **b**, *eat-2* mutant with target gene RNAi (#p < 0.001 compared to N2, ***p < 0.001 versus control RNAi, one-way ANOVA, n = 3 worm pellets). **c-e**, Expression of *ftn-1* with *tdh/F08F3.4* RNAi at the various mutants that are related to the regulation of *ftn-1* transcription. Worms were treated under **c**, normoxia (21% O_2), **d**, hypoxia (2% O_2), and **e**, hyperoxia (0.4M PQ) for 3 h (*p < 0.05, **p < 0.01, and ***p < 0.001 versus the control RNAi, one-way ANOVA, n =3 worm pellets). **f**, Quantification of Fe^{2+} /Fe^{3+} iron contents of nematode at intervals across lifespan (*p < 0.05, **p < 0.01, and ***p < 0.001 versus the vehicle group, one-way ANOVA, n = 3 worm pellets). **g-h**, Fe^{2+} /Fe^{3+} iron content levels of (**g**) DR or (**h**) *eat-2* mutants with the target gene RNAi were measured (#p < 0.001 compared to N2, **p < 0.01 and ***p < 0.001 versus control RNAi, one-way ANOVA, n =3 worm pellets). **i**, Relative Amplex Red fluorescence in supernatant of worms (*p < 0.05, **p < 0.01, and ***p < 0.001 versus control group, one-way ANOVA, n = 3 worm pellets). **j**, Total glutathione (GSH) level was normalized to the GSH level in worms not exposed to *tdh/F08F3.4* RNAi or threonine (*p < 0.05, **p < 0.01, and ***p < 0.001 versus control, one-way ANOVA, n = 3 worm pellets). **k**, Levels of the lipid peroxidation end product, malondialdehyde (MDA), were measured and normalized against the mean of untreated worms for independent samples (*p < 0.05 and **p < 0.05 versus control, one-way ANOVA, n = 3 worm pellets). **l-n**, Survival curves of ferrostatin-1 intervention with additional replicates. **o-q**, Survival curves of ftn-1 RNAi with 10 μM ferrostatin-1 administration with additional repeats. P-values listed in figure panel and survival data are presented in Supplementary Table 1. **r**, Relative Amplex Red fluorescence in supernatant of worms (***p < 0.001 versus control group, one-way ANOVA, n = 3 worm pellets). **s**, Total GSH level was

normalized to the GSH level in worms not exposed to *ftn-1* RNAi and ferrostatin-1 (***p < 0.001 versus control, one-way ANOVA, n = 3 worm pellets). **t**, Levels of the MDA were measured and normalized against the mean of untreated worms for independent samples (***p < 0.001 versus control, one-way ANOVA, n = 3 worm pellets).

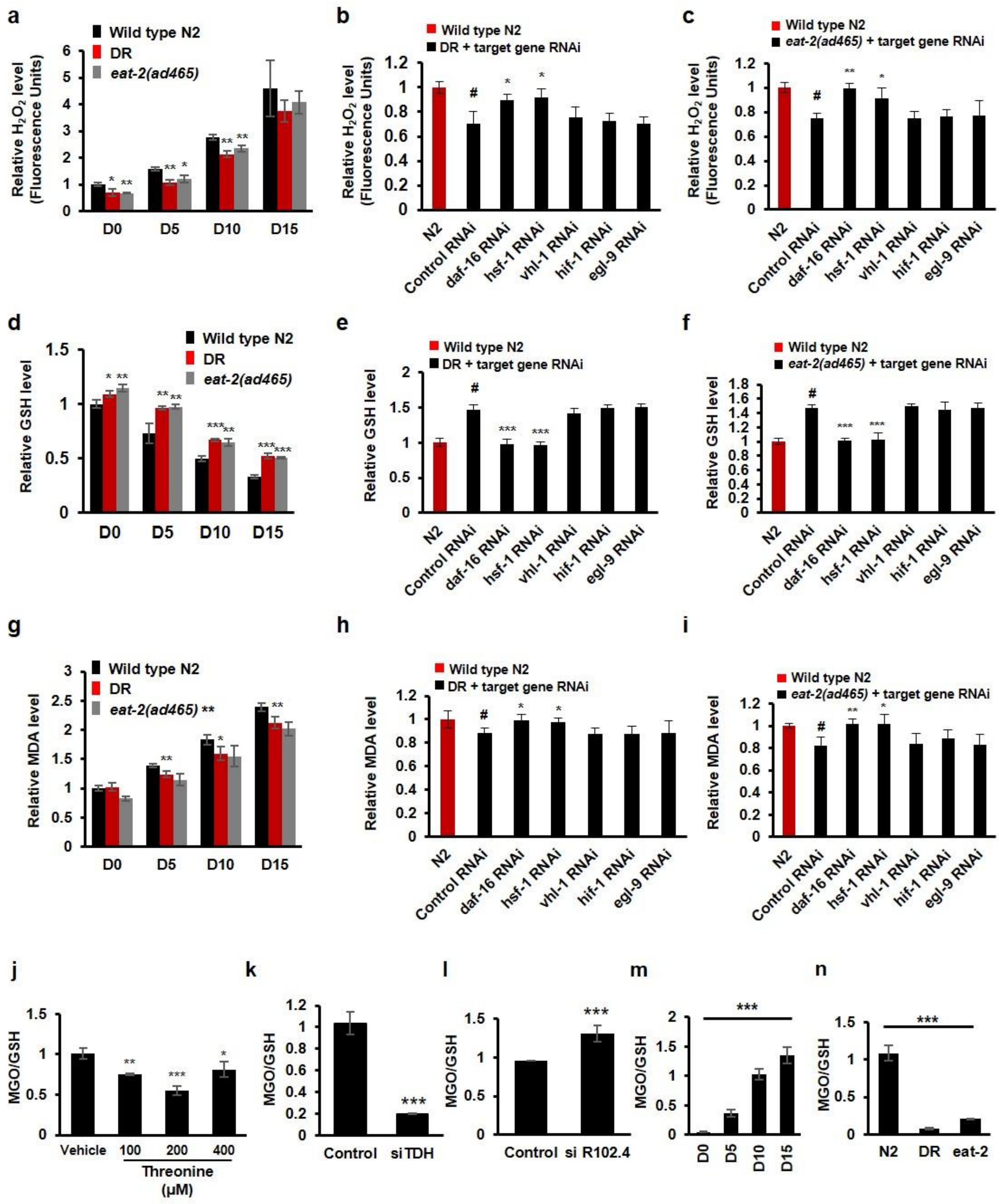

a
Wild type N2
DR
eat-2(ad465)
Relative H_2O_2 level (Fluorescence Units)
D0 D5 D10 D15
b
Wild type N2
DR + target gene RNAi
N2 Control RNAi daf-16 RNAi hsf-1 RNAi vhl-1 RNAi hif-1 RNAi egl-9 RNAi
c
Wild type N2
eat-2(ad465) + target gene RNAi
d
Relative GSH level
e
f
g
Relative MDA level
h
i
j
MGO/GSH
Vehicle 100 200 400
Threonine (μM)
k
Control siTDH
l
Control si R102.4
m
D0 D5 D10 D15
n
N2 DR eat-2

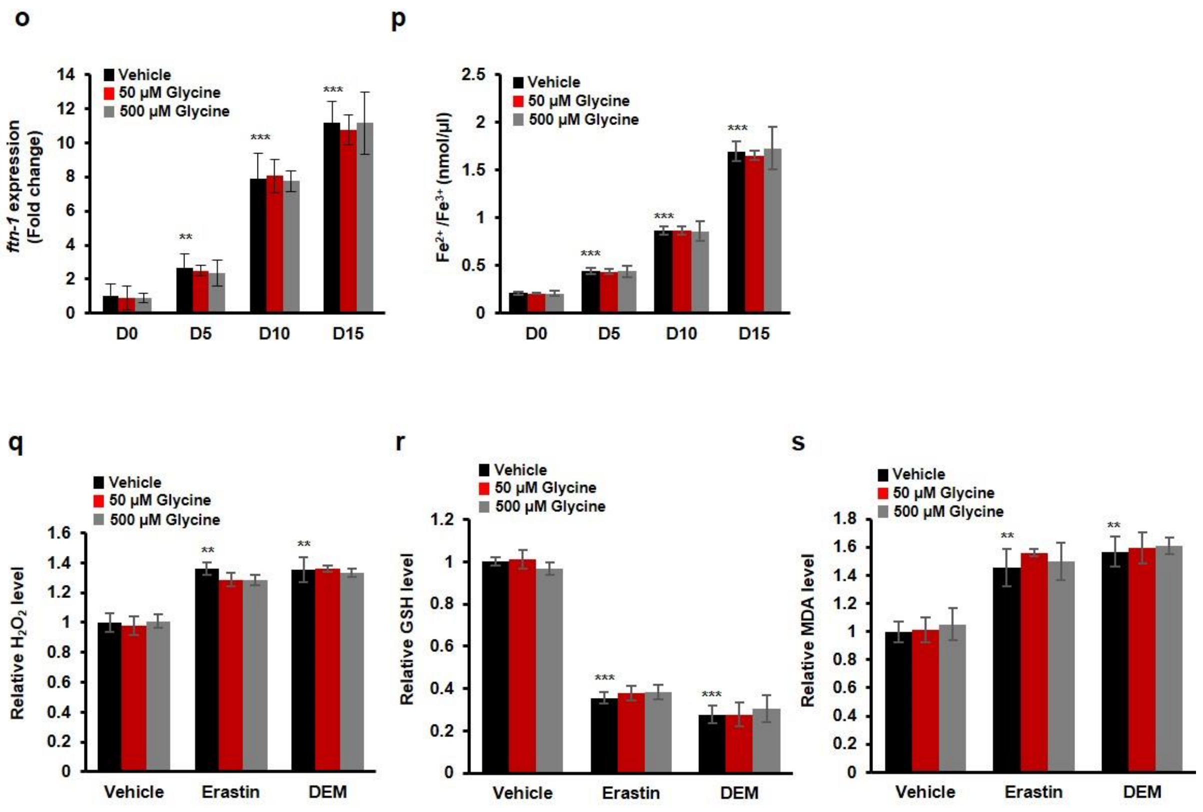

o
Vehicle
50 μM Glycine
500 μM Glycine
ftn-1 expression (Fold change)
D0
D5
D10
D15
p
Fe2+ /Fe3+ (nmol/μl)
q
Relative H2O2 level
Vehicle
Erastin
DEM
r
Relative GSH level
s
Relative MDA level

Supplementary Fig.8 | DR represented decreased ferroptosis in the manner of DAF-16 and HSF-1 and increasing threonine lowered age-associated factors. a, Relative Amplex Red fluorescence in supernatant of worm strains at intervals across lifespan (*p < 0.05 and **p < 0.01 versus the N2 group, one-way ANOVA, n = 3 worm pellets). **b-c**, Relative ROS level of (**b**) DR and (**c**) *eat-2* mutant with target gene RNAi (#p < 0.001 compared to N2, *p < 0.05 and **p < 0.01 versus the control RNAi, one-way ANOVA, n = 3 worm pellets). **d**, Relative GSH levels of worm strains at intervals across lifespan (*p < 0.05, **p < 0.01, and ***p < 0.001 versus N2, one-way ANOVA, n = 3 worm pellets). **e-f**, Relative GSH level of (**e**) DR and (**f**) *eat-2* mutant with target gene RNAi (#p < 0.001 compared to N2, ***p < 0.001 versus the control RNAi, one-way ANOVA, n = 3 worm pellets). **g**, Relative MDA levels of worm strains at intervals across lifespan (*p < 0.05 and **p < 0.01 versus N2, one-way ANOVA, n = 3 worm pellets). **h-i**, Relative MDA in (**h**) DR and (**i**) *eat-2* mutant with target gene RNAi (#p < 0.001 compared to N2, *p < 0.05 and **p < 0.01 versus control the RNAi, one-way ANOVA, n = 3 worm pellets). **j-n**, MGO/GSH levels under (**j**) threonine treatment (*p < 0.05, **p < 0.01, and ***p < 0.001 versus the vehicle, one-way ANOVA, n = 3 worm pellet), (**k**) *tdh/F08F3.4* RNAi, (**l**) *R102.4* RNAi (***p < 0.001 versus control RNAi, Student's t-test, n =3 worm pellets), (**m**) interval time points (***p < 0.001 versus Day 0, one-way ANOVA, n = 3 worm pellet), and (**n**) N2, DR, and *eat-2* mutant (***p < 0.001 versus N2, one-way ANOVA, n = 3 worm pellet). **o**, Expression of ftn-1 with 50 and 500 μM of glycine treatment (**p < 0.01, and ***p < 0.001 versus the day 0 vehicle, one-way ANOVA, n =3 worm pellets). **p**, Quantification of Fe^{2+} /Fe^{3+} iron contents of nematode at intervals across lifespan with glycine treatment (***p < 0.001 versus the day 0 vehicle group, one-way ANOVA, n = 3 worm pellets). **q**, Relative Amplex Red fluorescence in supernatant of worms (**p < 0.01, versus day 0 vehicle group, one-

way ANOVA, n = 3 worm pellets). **r**, Total glutathione (GSH) level was normalized to the GSH level in worms not exposed to glycine (***p < 0.001 versus vehicle, one-way ANOVA, n = 3 worm pellets). **s**, Levels of the MDA were measured and normalized against the mean of untreated worms for independent samples (***p < 0.001 versus vehicle, one-way ANOVA, n = 3 worm pellets). Error bars represent the mean ± s.d.

Supplementary Table 1 | Summary of lifespan data

Strain	Treatment	Mean lifespan (days) ± SEM		% difference	P-value	n (animals)	Figure
N2	Threonine (µM)	Vehicle	15.7 ± 0.3			90	Fig. 1c
		100	17.2 ± 0.4	9.5	0.01318723	90	
		200	18.5 ± 0.4	18	9.818E-06	87	
		400	16.5 ± 0.3	5.4	0.02632925	89	
		800	15.1 ± 0.3	-3.5	0.29197009	88	
		1000	15.5 ± 0.3	-1.3	0.47151619	90	
		2000	15.4 ± 0.3	-1.9	0.39110771	83	
N2	Threonine (µM)	Vehicle	15.7 ± 0.3			90	Supplementary Fig. 1a
		100	17.4 ± 0.4	10.8	0.025	92	
		200	18.4 ± 0.4	17.3	4.0351E-05	92	
		400	16.5 ± 0.3	5	0.03624563	89	
		800	15.2 ± 0.3	-3.1	0.24118061	93	
		1000	15.4 ± 0.3	-1.9	0.24921714	91	
		2000	15.6 ± 0.3	-0.8	0.29940049	91	
N2	Threonine (µM)	Vehicle	15.8 ± 0.3			89	Supplementary Fig. 1b
		100	17.1 ± 0.4	8.7	0.01828145	91	
		200	18.2 ± 0.4	15.7	2.8716E-05	96	
		400	16.9 ± 0.3	7.4	0.02617666	96	
		800	15.9 ± 0.3	0.9	0.60101601	95	
		1000	15.5 ± 0.3	-1.8	0.695817	93	
		2000	15.3 ± 0.3	-2.7	0.56453947	93	
N2	Threonine (mM)	Vehicle	15.3 ± 0.2			92	Supplementary Fig.1h
		10	7.1 ± 0.2	8.8	0.03367001	93	
		20	8.1 ± 0.3	16.5	0.00012714	108	
		40	7.6 ± 0.2	-11.6	0.02688507	93	
N2	Threonine (mM)	Vehicle	15.4 ± 0.3			106	Supplementary Fig.1i
		10	7.5 ± 0.3	8.3	0.09166065	98	
		20	7.5 ± 0.3	18.4	1.5278E-05	110	
		40	8.0 ± 0.4	-9.6	0.01066835	93	
N2	Threonine (mM)	Vehicle	15.9 ± 0.2			98	Supplementary Fig.1j
		10	7.4 ± 0.2	7.2	0.18277221	105	
		20	7.4 ± 0.2	13.5	0.00141251	115	
		40	7.0 ± 0.2	-11.3	0.03745759	107	
N2	Control RNAi		14.6 ± 0.3			92	2k
	TDH RNAi		19.5 ± 0.4	33.4	4.1701E-14	96	

N2	Control RNAi		15.8 ± 0.3			88	Supplementary Fig. 2b
	TDH RNAi		21.5 ± 0.5	36.1	1.5979E-09	90	
N2	Control RNAi		15.2 ± 0.3			91	Supplementary Fig. 2c
	TDH RNAi		20.3 ± 0.4	33.6	3.5476E-11	94	
N2	Control RNAi		15.6 ± 0.3			96	Supplementary Fig. 3a
	R102.4 RNAi		13.0 ± 0.2	-16.8	0.00015631	94	
N2	Control RNAi		15.7 ± 0.3			90	Supplementary Fig. 3b
	R102.4 RNAi		13.1 ± 0.2	-16.5	3.6303E-06	95	
N2	Control RNAi		14.7 ± 0.3			90	Supplementary Fig. 3c
	R102.4 RNAi		12.7 ± 0.3	-13.6	0.00875291	95	
eat-2(ad1116)	Control RNAi		21.7 ± 0.4			102	3d
	TDH RNAi		21.6 ± 0.4	-0.4	0.785831	97	
eat-2(ad1116)	Control RNAi		21.8 ± 0.4			94	Supplementary Fig. 4d
	TDH RNAi		21.2 ± 0.5	-2.6	0.97644574	94	
eat-2(ad1116)	Control RNAi		21.4 ± 0.5			93	Supplementary Fig. 4e
	TDH RNAi		20.9 ± 0.5	-2.3	0.42830439	96	
eat-2(ad1116)	Threonine (μM)	Vehicle	21.3 ± 0.4			91	Supplementary Fig. 5a
		100	21.1 ± 0.4	-0.7	0.82883163	91	
		200	20.4 ± 0.3	-3.8	0.19526359	88	
		400	20.7 ± 0.4	-2.9	0.91731597	88	
eat-2(ad1116)	Threonine (μM)	Vehicle	21.0 ± 0.3			98	Supplementary Fig. 5b
		100	21.2 ± 0.4	0.8	0.47588931	91	
		200	20.4 ± 0.3	-2.8	0.3479877	94	
		400	20.5 ± 0.4	-2.3	0.69626906	97	
eat-2(ad1116)	Threonine (μM)	Vehicle	21.0 ± 0.4			96	Supplementary Fig. 5c
		100	21.0 ± 0.4	0.3	0.61436981	90	
		200	20.4 ± 0.3	-2.7	0.31910299	94	
		400	21.0 ± 0.7	-2.6	0.78454987	93	
daf-2(e1370)	Control RNAi		22.5 ± 0.5			93	3e
	TDH RNAi		25.9 ± 0.6	15.1	0.00024409	97	
daf-2(e1370)	Control RNAi		22.2 ± 0.5			96	Supplementary Fig. 4f
	TDH RNAi		25.8 ± 0.6	16.2	0.00021323	96	
daf-2(e1370)	Control RNAi		22.3 ± 0.5			95	

	TDH RNAi		25.5 ± 0.6	14.6	0.00222086	102	Supplementary Fig. 4g
daf-2(e1370)			22.6 ± 0.5			92	Supplementary Fig. 5d
	Threonine (μM)	100	25.1 ± 0.5	10.9	0.01662628	97	
		200	25.3 ± 0.6	11.7	0.00382294	100	
		400	22.2 ± 0.5	-1.8	0.87212671	96	
daf-2(e1370)			22.3 ± 0.5			92	Supplementary Fig. 5e
	Threonine (μM)	100	24.5 ± 0.5	9.5	0.05007683	94	
		200	25.2 ± 0.5	12.6	0.00273856	97	
		400	21.9 ± 0.5	-2.1	0.70293411	94	
daf-2(e1370)			22.7 ± 0.5			89	Supplementary Fig. 5f
	Threonine (μM)	100	24.5 ± 0.6	8	0.03400012	86	
		200	25.0 ± 0.5	10.4	0.01544843	92	
		400	21.9 ± 0.5	-3.2	0.83582142	88	
daf-16(mu86)	Control RNAi		13.0 ± 0.3			102	3f
	TDH RNAi		13.5 ± 0.3	0.9	0.62725723	99	
daf-16(mu86)	Control RNAi		12.9 ± 0.3			97	Supplementary Fig. 4h
	TDH RNAi		13.5 ± 0.3	4.5	0.36382365	95	
daf-16(mu86)	Control RNAi		12.8 ± 0.3			93	Supplementary Fig. 4i
	TDH RNAi		12.8 ± 0.3	0.6	0.79278221	92	
daf-16(mu86)			12.9 ± 0.3			87	Supplementary Fig. 5g
	Threonine (μM)	100	13.3 ± 0.3	2.9	0.48525786	89	
		200	13.7 ± 0.3	6.4	0.16755809	87	
		400	13.2 ± 0.3	1.8	0.34965062	90	
daf-16(mu86)			12.7 ± 0.3			92	Supplementary Fig. 5h
	Threonine (μM)	100	13.2 ± 0.3	4.6	0.50873332	97	
		200	12.7 ± 0.3	0.5	0.50153425	93	
		400	12.5 ± 0.4		0.85684873	94	
daf-16(mu86)			12.3 ± 0.3			93	Supplementary Fig. 5i
	Threonine (μM)	100	13.0 ± 0.3	5	0.32790939	95	
		200	12.7 ± 0.3	3.2	0.4104227	95	
		400	12.2 ± 0.3	-1	0.65040023	92	
hsf-1 (sy441)	Control RNAi		8.0 ± 0.3			80	3g
	TDH RNAi		8.2 ± 0.3	2.7	0.49201391	83	
hsf-1 (sy441)	Control RNAi		7.8 ± 0.3			86	Supplementary Fig. 4j
	TDH RNAi		7.8 ± 0.2	1	0.83963669	90	
hsf-1 (sy441)	Control RNAi		7.2 ± 0.2			91	

	TDH RNAi		7.2 ± 0.3	1	0.94061687	89	Supplementary Fig. 4k
hsf-1 (sy441)			7.5 ± 0.2			98	
hsf-1 (sy441)		100	7.1 ± 0.2	-5.7	0.54254055	98	Supplementary Fig. 5j
hsf-1 (sy441)	Threonine (μM)	200	8.1 ± 0.3	7.6	0.28327525	98	
hsf-1 (sy441)		400	7.6 ± 0.2	0.9	0.28874204	99	
hsf-1 (sy441)			7.9 ± 0.3			86	
hsf-1 (sy441)		100	7.5 ± 0.3	0.9	0.58359758	95	Supplementary Fig. 5k
hsf-1 (sy441)	Threonine (μM)	200	7.5 ± 0.3	-5.1	0.54965683	89	
hsf-1 (sy441)		400	8.0 ± 0.4	2	0.70293555	96	
hsf-1 (sy441)			7.2 ± 0.2			81	
hsf-1 (sy441)		100	7.4 ± 0.2	3.3	0.4769379	90	Supplementary Fig. 5l
hsf-1 (sy441)	Threonine (μM)	200	7.4 ± 0.2	2.9	0.38189565	93	
hsf-1 (sy441)		400	7.0 ± 0.2	-3.1	0.42616027	99	
N2	Control RNAi		14.8 ± 0.3			88	4a
	gst-19 RNAi		16.3 ± 0.4	18.1	0.00011133	87	
N2	Control RNAi		15.3 ± 0.3			87	Supplementary Fig. 6a
	gst-19 RNAi		18.5 ± 0.5	21.1	1.5979E-09	90	
N2	Control RNAi		15.6 ± 0.3			90	Supplementary Fig. 6b
	gst-19 RNAi		17.7 ± 0.4	13.8	0.01044407	89	
N2	Control RNAi		15.7 ± 0.3			91	4b
	T12D8.5 RNAi		20.5 ± 0.5	13.2	0.01854408	95	
N2	Control RNAi		15.5 ± 0.2			91	Supplementary Fig. 6c
	T12D8.5 RNAi		19.8 ± 0.5	21.2	6.7349E-09	92	
N2	Control RNAi		15.3 ± 0.3			85	Supplementary Fig. 6d
	T12D8.5 RNAi		20.6 ± 0.4	15.6	0.00980001	88	
N2	Control RNAi		18.4 ± 0.4			85	4c
	cnc-2 RNAi		20.5 ± 0.5	11.1	0.02717264	87	
N2	Control RNAi		18.2 ± 0.4			89	Supplementary Fig. 6e
	cnc-2 RNAi		20.4 ± 0.4	12.1	0.00148891	91	
N2	Control RNAi		18.0 ± 0.4			85	Supplementary Fig. 6f
	cnc-2 RNAi		20.6 ± 0.4	14.4	0.03255776	88	
N2	Control RNAi		14.0 ± 0.2			92	4d
	ftn-1 RNAi		14.0 ± 0.3	-0.2	0.75088368	89	

N2	Control RNAi		14.0 ± 0.3			90	Supplementary Fig. 6g
	ftn-1 RNAi		14.3 ± 0.3	2.4	0.60254214	90	
N2	Control RNAi		14.0 ± 0.2			95	Supplementary Fig. 6h
	ftn-1 RNAi		13.8 ± 0.2	-1.4	0.90572168	94	
ftn-1 (ok3625)	Control RNAi		14.5 ± 0.3			100	4e
	TDH RNAi		14.5 ± 0.3	0.3	0.82261213	102	
ftn-1 (ok3625)	Control RNAi		14.6 ± 0.3			93	Supplementary Fig. 6i
	TDH RNAi		14.5 ± 0.3	-0.6	0.9317674	92	
ftn-1 (ok3625)	Control RNAi		14.6 ± 0.3			96	Supplementary Fig. 6j
	TDH RNAi		14.5 ± 0.3	-0.1	0.68241871	95	
ftn-1 (ok3625)			14.5 ± 0.3			89	4f
	Threonine (μM)	100	14.5 ± 0.3	-0.38	0.83269299	91	
		200	14.4 ± 0.3	-1.66	0.8348663	95	
		400	14.6 ± 0.4	0.5	0.89492254	92	
ftn-1 (ok3625)			14.6 ± 0.3			93	Supplementary Fig. 6k
	Threonine (μM)	100	14.5 ± 0.3	-0.6	0.86549436	92	
		200	14.3 ± 0.3	-1.6	0.50980404	92	
		400	14.4 ± 0.3	-1.4	0.68673723	92	
ftn-1 (ok3625)			14.5 ± 0.3			92	Supplementary Fig. 6l
	Threonine (μM)	100	14.5 ± 0.3	-0.4	0.38539193	95	
		200	14.9 ± 0.3	2.4	0.92239689	92	
		400	14.9 ± 0.4	2.9	0.74442601	93	
N2	Ferrostatin-1 (μM)	Vehicle	15.5 ± 0.3			97	Supplementary Fig.7l
		1	15.9 ± 0.3	5.9	0.340305277	102	
		10	8.1 ± 0.3	6.5	0.075836973	89	
		100	7.6 ± 0.2	11	0.001743325	126	
N2	Ferrostatin-1 (μM)	Vehicle	15.4 ± 0.3			103	Supplementary Fig.7m
		1	16.2 ± 0.3	5.3	0.135666999	120	
		10	7.5 ± 0.3	8	0.076057204	103	
		100	8.0 ± 0.4	13	0.001860262	118	
N2	Ferrostatin-1 (μM)	Vehicle	15.5 ± 0.3			108	Supplementary Fig.7n
		1	16.3 ± 0.3	4.8	0.114294978	104	
		10	7.4 ± 0.2	7	0.082703034	85	
		100	7.0 ± 0.2	13	7.95786E-05	116	
N2	ftn-1 RNAi	Vehicle	14.2 ± 0.2			103	Supplem

		10 μM ferrostatin	14.4 ± 0.3	1.9	0.972514401	91	entary Fig.7o
N2	ftn-1 RNAi	Vehicle	14.2 ± 0.1			88	Supplementary Fig.7p
		10 μM ferrostatin	14.1 ± 0.2	-0.9	0.863282627	100	
N2	ftn-1 RNAi	Vehicle	14.3 ± 0.2			86	Supplementary Fig.7q
		10 μM ferrostatin	14.3 ± 0.3	-0.6	0.663675467	93	

Supplementary Table 2 | Sequences of qPCR primers

Primer	Sequence_Fwd	Sequence_Rev
tdh	TCGCGTTAACGCTAGCATGGATCTC	GTAACATCAGAGATTTTGAGACAC
R102.4	GGCGAGGAGATAATCGTCGG	GTGACAATCGGGTATACTCGTCA
gst-19	TCGCGTTAACGCTAGCATGGATCTC	GTAACATCAGAGATTTTGAGACAC
T12D8.5	TCGCGTTAACGCTAGCATGGATCTC	GTAACATCAGAGATTTTGAGACAC
cnc-2	TCGCGTTAACGCTAGCATGGATCTC	GTAACATCAGAGATTTTGAGACAC
ftn-1	TCGCGTTAACGCTAGCATGGATCTC	GTAACATCAGAGATTTTGAGACAC
daf-16	GCGAATCGGTTCCAGCAATTCCAA	ATCCACGGACACTGTTCAACTCGT
hsf-1	GGAAAGTGGTCCACATCGAG	TTCACTCTCCCGCAGGATGG
hif-1	CAGTGATTCTTCAATTCTTTACGTC	GGATTAACACAGACAGATTTAACAG
egl-9	GCCGACTTTCAATCCACTTC	AATGATCGGAGATCGACTGG
actin	GAGAGGGAAATCGTGCGTGAC	CATCTGCTGGAAGGTGGACA
cdc-42	CTGCTGGACAGGAAGATTACG	CTCGGACATTCTCGAATGAAG
Y45F10D.4	GTCGCTTCAAATCAGTTCAG	GTTCTTGTCAAGTGATCCGACA
