# Supplementary material for: L-threonine promotes healthspan by expediting ferritin-dependent ferroptosis inhibition in C. elegans
Source: Nat Commun. 2022 Nov 2;13:6554. doi: 10.1038/s41467-022-34265-x (PMC9628521; doi:10.1038/s41467-022-34265-x)
Supplement: Supplementary file 3 — Reporting Summary [file 41467_2022_34265_MOESM3_ESM.pdf]

nature portfolio

Corresponding author(s): Juewon Kim and Dongryeol Ryu

Last updated by author(s): Aug 4, 2022

Reporting Summary

Nature Portfolio wishes to improve the reproducibility of the work that we publish. This form provides structure for consistency and transparency in reporting. For further information on Nature Portfolio policies, see our Editorial Policies and the Editorial Policy Checklist.

Statistics

For all statistical analyses, confirm that the following items are present in the figure legend, table legend, main text, or Methods section.

n/a	Confirmed	
☐	☒	The exact sample size (*n*) for each experimental group/condition, given as a discrete number and unit of measurement
☐	☒	A statement on whether measurements were taken from distinct samples or whether the same sample was measured repeatedly
☐	☒	The statistical test(s) used AND whether they are one- or two-sided<br>*Only common tests should be described solely by name; describe more complex techniques in the Methods section.*
☒	☐	A description of all covariates tested
☐	☒	A description of any assumptions or corrections, such as tests of normality and adjustment for multiple comparisons
☐	☒	A full description of the statistical parameters including central tendency (e.g. means) or other basic estimates (e.g. regression coefficient) AND variation (e.g. standard deviation) or associated estimates of uncertainty (e.g. confidence intervals)
☐	☒	For null hypothesis testing, the test statistic (e.g. *F*, *t*, *r*) with confidence intervals, effect sizes, degrees of freedom and *P* value noted<br>*Give P values as exact values whenever suitable.*
☒	☐	For Bayesian analysis, information on the choice of priors and Markov chain Monte Carlo settings
☐	☒	For hierarchical and complex designs, identification of the appropriate level for tests and full reporting of outcomes
☒	☐	Estimates of effect sizes (e.g. Cohen's *d*, Pearson's *r*), indicating how they were calculated

Our web collection on statistics for biologists contains articles on many of the points above.

Software and code

Policy information about availability of computer code

Data collection	All microscopy images were acquired using the Zen blue software (Ver. 3.4, Carl Zeiss).
Data analysis	Statistical analysis of the automated worm tracking videos by TSView 7 (OnFocus Laboratories) was performed as described in the text. Statistical analysis of RNA-seq comparison was performed using DESeq2 as previously described. All microscopy fluorescence quantifications were done in the Zen software (Carl Zeiss). All statistical tests for fluorescence quantifications and behavior assays were conducted using Zen blue (Carl Zeiss) and Excel as described in figure legends.

For manuscripts utilizing custom algorithms or software that are central to the research but not yet described in published literature, software must be made available to editors and reviewers. We strongly encourage code deposition in a community repository (e.g. GitHub). See the Nature Portfolio guidelines for submitting code & software for further information.

Data

Policy information about availability of data

All manuscripts must include a data availability statement. This statement should provide the following information, where applicable:

- Accession codes, unique identifiers, or web links for publicly available datasets
- A description of any restrictions on data availability
- For clinical datasets or third party data, please ensure that the statement adheres to our policy

All data are available within this Article and its Supplementary Information. Original data points in graphs are shown in Source Data files. Raw and processed RNA-seq data is available at NCBI's SRA accession #PRJNA772096.

Field-specific reporting

Please select the one below that is the best fit for your research. If you are not sure, read the appropriate sections before making your selection.

☒ Life sciences ☐ Behavioural & social sciences ☐ Ecological, evolutionary & environmental sciences

For a reference copy of the document with all sections, see nature.com/documents/nr-reporting-summary-flat.pdf

Life sciences study design

All studies must disclose on these points even when the disclosure is negative.

Sample size	Sample size was determined based on experimental trials and previous publications on similar experiments. No statistical methods were used to predetermine sample size.
Data exclusions	No data were excluded.
Replication	All experiments were successfully repeated at least two or three times, and the number of independent experiments or biological replicates is indicated in the figure legends. At each repeat, all control and experimental conditions were included, and the results of all independent experiments were combined.
Randomization	Whenever possible, all samples/animals were randomized.
Blinding	Whenever possible for behavioral analysis, the scoring was done blindly. For all other molecular and microscopy experiments, experimenters were not blinded to the allocation during experiments as it does not include clinical trials.

Reporting for specific materials, systems and methods

We require information from authors about some types of materials, experimental systems and methods used in many studies. Here, indicate whether each material, system or method listed is relevant to your study. If you are not sure if a list item applies to your research, read the appropriate section before selecting a response.

Materials & experimental systems

n/a	Involved in the study	
☒	☐	Antibodies
☒	☐	Eukaryotic cell lines
☒	☐	Palaeontology and archaeology
☐	☒	Animals and other organisms
☒	☐	Human research participants
☒	☐	Clinical data
☒	☐	Dual use research of concern

Methods

n/a	Involved in the study	
☒	☐	ChIP-seq
☒	☐	Flow cytometry
☒	☐	MRI-based neuroimaging

Animals and other organisms

Policy information about studies involving animals; ARRIVE guidelines recommended for reporting animal research

Laboratory animals	C. elegans. Whenever synchronization of developmental stages was necessary, animals were egg prepped according to standard protocol and synchronized at the L1 stage. They were then plated on food and collected after 8 +/-1 hrs, 21 +/-1 hrs, 30 +/- 2hr, 40 +/- 2hrs, and 53 +/- 2hrs for L1, L2, L3, L4, and adult stages, respectively for either molecular or behavioral analysis. These time points were chosen such that the animals were in the middle of each larval stage or relatively early in adulthood for the analysis.
Wild animals	No wild animals used.
Field-collected samples	No field-collected samples were used.
Ethics oversight	No ethics approval was necessary.

Note that full information on the approval of the study protocol must also be provided in the manuscript.
